# Recovery of Aging-Related Size Increase of Skin Epithelial Cells: *In vivo* Mouse and *In vitro* Human Study

**DOI:** 10.1371/journal.pone.0122774

**Published:** 2015-03-25

**Authors:** Igor Sokolov, Natali V. Guz, Swaminathan Iyer, Amy Hewitt, Nina A. Sokolov, Joseph S. Erlichman, Craig D. Woodworth

**Affiliations:** 1 Departments of Mechanical Engineering, Biomedical Engineering, Physics, Tufts University, Medford, Massachusetts, United States of America; 2 Department of Chemistry, Clarkson University, Potsdam, New York, United States of America; 3 School of Chemistry and Biochemistry, The University of Western Australia, Crawley, WA, Australia; 4 Department of Biology, Queen's University, Kingston, Ontario, Canada; 5 Department of Biology, Clarkson University, Potsdam, New York, United States of America; 6 Department of Biology, St. Lawrence University, Canton, New York, United States of America; University of Waterloo, CANADA

## Abstract

The size increase of skin epithelial cells during aging is well-known. Here we demonstrate that treatment of aging cells with cytochalasin B substantially decreases cell size. This decrease was demonstrated on a mouse model and on human skin cells *in vitro*. Six nude mice were treated by topical application of cytochalasin B on skin of the dorsal left midsection for 140 days (the right side served as control for placebo treatment). An average decrease in cell size of 56±16% resulted. A reduction of cell size was also observed on primary human skin epithelial cells of different *in vitro* age (passages from 1 to 8). A cell strain obtained from a pool of 6 human subjects was treated with cytochalasin B *in vitro* for 12 hours. We observed a decrease in cell size that became statistically significant and reached 20–40% for cells of older passage (6–8 passages) whereas no substantial change was observed for younger cells. These results may be important for understanding the aging processes, and for cosmetic treatment of aging skin.

## Introduction

Biological aging is a complex process [1] which affects many biophysical parameters of cells [2, 3]. The increase of rigidity of human epithelial tissues with ageing [4] has been implicated in the pathogenesis of many diseases associated with aging including vascular diseases, kidney disease, cataracts, Alzheimer's Dementia [5],[6], complications of diabetes, and cardiomyopathies [7]. Although the dermis layer changes substantially, epithelial cells were shown to become considerably more rigid after aging *in vitro* [8]. Another well-known effect of aging results in an increase in size of skin corneocytes [3, 9–12].

Recently, it was shown that the age—related increase of rigidity of epithelial skin cells was associated with an increase in density of F-actin fibers of the cytoskeleton. A treatment was suggested for age-related loss of elasticity in epithelial tissues using cytochalasins [13]. Cytochalasins are cell permeable fungal toxins which bind to the barbed end of actin filaments inhibiting both the association and dissociation of its subunits. The action of cytochalasins was shown *in vitro* to result in a decrease of the elastic modulus of old cells back to the level of young cells [13].

Here we report on another useful action of cytochalasin B, a decrease of the size of skin epithelial cells. These results may be important for better understanding the aging processes in cells. To show it *in vivo*, we use a mouse model. To demonstrate a similar behavior of human skin epithelial cells, we use a mixture of primary human epithelial cells derived from skin of six healthy donors. In both cases, we observed a significant reduction of cell size. In the case of mouse skin, the skin treated with cytochalasin B looked smoother compared to that treated with placebo. Histological analysis of mouse skin after the end of the experiment (close to the natural lifetime limit of these mice) showed no visible cell or tissue abnormalities. The results of this work may be interesting for cosmetic treatment of visual sign s of aging [14]. From a fundamental point of view, aging of skin may be a model for biological aging of other organs [15].

## Materials and Methods

### Mice samples and cytochalasin B treatment

Nude mice (semi-compromised immune system, NU/J) of either sex aged four-weeks (n = 10) were obtained from Jackson Laboratories. To avoid the stress, the animals were grown in cages (open to ambient conditions) for several months. Six mice were used in the study of cytochalasin treatment, and four mice remained untreated as a control group. All cages were in a specially designated licensed animal facility of Saint Lawrence University.

To study the action of cytochalasin B (Sigma Aldrich, St. Louis, MO), a carrier cream was made as follows. Cytochalasin was added to a base cream (jojoba oil based, New Directions Aromatics, USA) at the concentration of 1 ug/g cream. This cream was topically applied to three sites (cheek pouch, dorsal midsection, dorsal/posterior above hip) on the left side of the mouse’s body to test the cream action for possible irritation or inflammation. The right side of the mouse served as a control. The cream applied to the right side of the animal was identical to the test cream without the active ingredient, cytochalasin B. Mouse dorsal midsections were used for the long-term study. Each mouse had both treated and untreated sides, thus it served as its own control. Both active drug and placebo carrier creams were applied to each animal twice a day for the duration of 140 days. To verify the absence of subcutaneous abnormalities due to cytochalasin B treatment, the mice were sacrificed by rapid decapitation using a guillotine at the end of the experiment (which is almost coincided with the projected lifespan of mice with semi-compromised system, 1 year).

The Institutional Animal Care and Use Committee of Saint Lawrence University approved this study. The procedure of rapid decapitation was designed to assure that discomfort and pain to animals was limited to that which was unavoidable for the conduct of scientifically valuable research. This included the use of analgesic and tranquillizing drugs where indicated and appropriate to minimize discomfort and pain to the animals. Reasonable precautions were taken in the approved study. If any animal developed a rash or exhibited serious epidermal inflammation during the course of treatment, the experiment was terminated and the concentration of the cytochalasin administered reassessed. Rapid decapitation of mice is an acceptable form of euthanasia as stated in the animal care guidelines put forth by the National Institute of Health.

Mouse skin tissues were harvested from the test sites and the contralateral control sites and were maintained in tissue culture media immediately after the extraction. The cross-sections were stained with Neutral Red, and 30 um cryostat sections were examined by optical microscopy. To enhance confocal fluorescent contrast of self-fluorescent skin, sections were immersed in aqueous solution (1uM) of Rhodamine 6G dye for 15 minutes and subsequently rinsed with clean water right before the imaging.

### Cell culture and cytochalasin B treatment

Primary cultures of foreskin epithelial cells collected from six human subjects were prepared by a two-stage enzymatic digestion as described [16] and cells were maintained in keratinocyte serum free medium (Invitrogen, Carlsbad, CA) to avoid contamination of epithelial cells with fibroblasts. Young cells started from passage 1 (cells were adapted to culture within one week after removal from liquid nitrogen). Cells were aged *in vitro* up to passage 8 (over 50 population doublings). The treatment of cells with cytochalasin B (Sigma Aldrich, St. Louis, MO) was done with 5 μg/mL solution of cytochalasin B in the growth medium for 12 hours.

All human tissue was obtained from the Cooperative Human Tissue Network (CHTN, http://www.chtn.nci.nih.gov). Written informed consent was obtained when collecting tissues from patients according to their published guidelines (http://chtn.nci.nih.gov/phspolicies.html). The purchased tissues were exempt from further IRB approval by Clarkson University since no personal information of the commercial samples was recorded and the samples were collected by the CHTN for other medical purposes according to the NIH published guidelines.

### Imaging of cells

#### Confocal microscopy of mouse skin

A C1 Eclipse Nikon confocal microscope built on the base of Nikon TE2000U inverted microscope was used for imaging skin samples prepared as described above. 10x (NA 0.15), 20x (NA 0.75) and 60x (NA 0.9) CFI APO objectives were used. Blue argon ion laser was utilized for excitation of fluorescence. Specifically, 488nm wavelength of light was used. Green fluorescent channel was recorded. The images are recorded with resolution of at least one megapixel.

#### Imaging of human epithelial cells

Nikon TE2000U inverted microscope with Hamamatsu digital camera was used to image cells in the Hoffman contrast mode. The cells were imaged after some relaxation time after trypsinization, without letting them adhere to culture dishes. The cells imaged were essentially just precipitated to the bottom of the culture dish. This was done to avoid confusion between the cell volume (what we study) and area (what we measure), which are not necessarily correlated if cells are attached to a flat surface. All of the cells were round (or close to round shape), and therefore, we could correlate the cell volume and area.

### Image processing

Confocal images of cells in mouse skin were processed manually. A square of 10x10 microns was moved over the images and the number of cells inside the square was calculated. The digital images of human epithelial cells collected in the Hoffman contrast mode were processed first through image-processing software (GIMP, GNU group) to adjust the contrast and brightness. Then the Image J (free image processing and analysis program by the University of Texas Health and Science Center, San Antonio) software package was used to process the images automatically, and calculate the area of individual cells. Standard ANOVA statistical tests were utilized.

## Results and Discussion

### Mouse in vivo model

**Fig 1:**
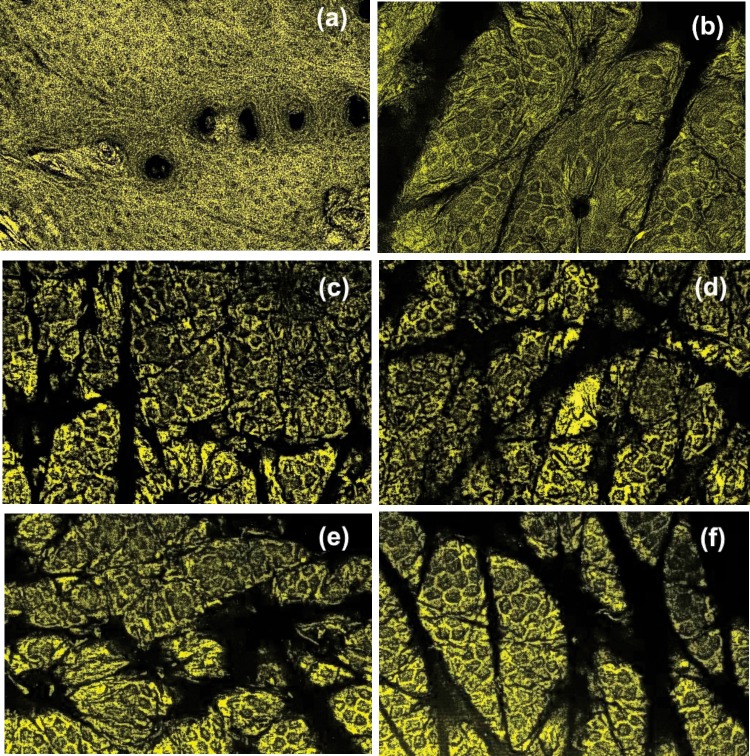
Representative confocal images of epithelial cells of mouse skin. Skin is shown treated with cytochalasin B (left) and with placebo (right). Skin samples of mouse #2 (a,b), 5 (c,d), and 1 (e,f) are shown.

**Fig 2:**
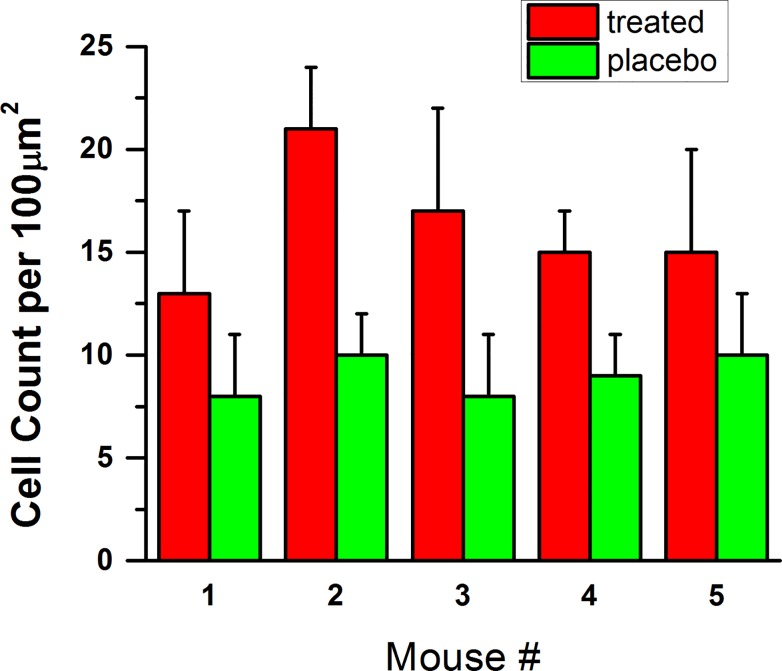
Number of cells per a definite area. The average numbers of cells within 10x10 micron^2 area as well as one standard deviation.

Visual review of the mouse skin condition showed no abnormalities before or after treatment. Representative confocal images of epithelial cells of mouse skin, both treated with cytochalasin B and with placebo are shown in Fig. 1 for mouse 1, 2, and 5. These mice represent the largest, smallest, and the middle change of the cell size (see Fig. 2 later). One can clearly see individual cells. The contrast is slightly lower for mouse 2 (Figs. 1A and B). This is presumably due to a thicker corneocyte layer.

The average numbers of cells calculated within the 10x10 micron^2^ area as well as one standard deviation are presented in Fig. 2 (mouse sample # 6 was damaged during preparation and is not shown). One can see that the cell size changed significantly (at the confidence level p<10^−6^) after treatment with cytochalasin.

**Fig 3:**
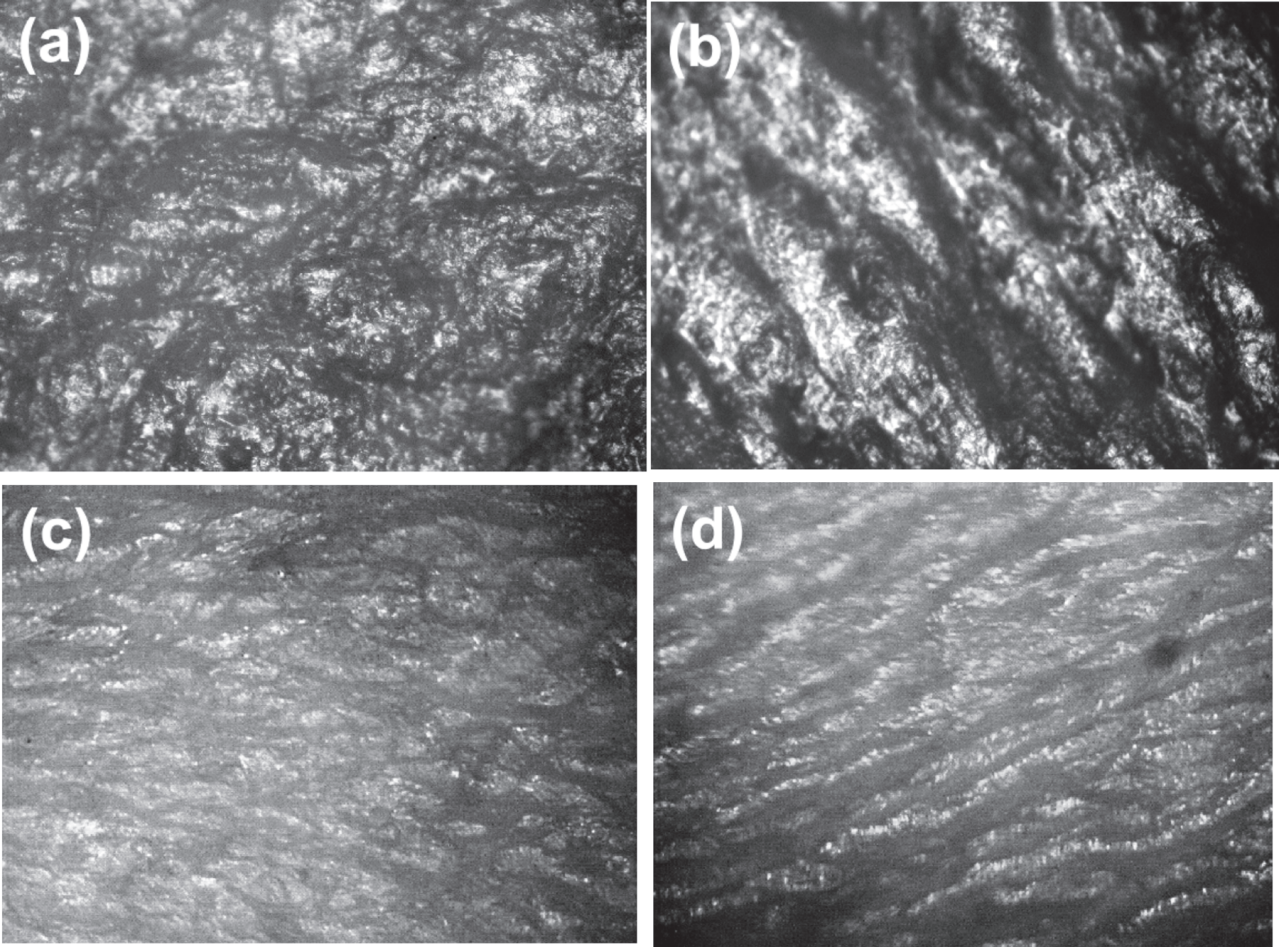
Representative bright field images of skin. (a,b) Samples for mouse 2; (cd) Samples for mouse 5 are shown. (a,c) Skin treated with cytochalasin B; and(b,d) with placebo cream.

Fig. 3 illustrates a brightfield view of the skin of mouse 2 and 5 collected from both skin treated with cytochalasin B and placebo cream. One can qualitatively see that the skin surface treated with cytochalasin B is smoother. This effect presumably comes from the geometrical decrease of the size of each cell, which can lead to some stretching of the skin surface. The overall decrease of the cell stiffness (reported on human cells [13] and observed on mouse skin (to be published)) may also contribute to a decreased amount of mechanical “buckling” seen on the untreated skin (Figs. 3C and D).

**Fig 4:**
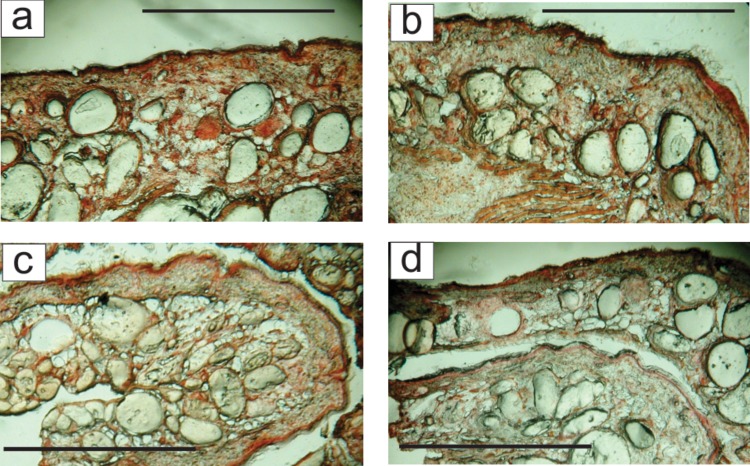
Example of cross-section of mouse skin. (c,d) mice #2 and (a,b) mice # 4. (a,c) Cytochalasin-treated skin; (b,d) The side treated with placebo. The scale bar is 100 microns.

As such, this result may be useful in cosmetic treatment of aging skin. However, it is important to know that cytochalasin B does not cause any undesirable changes inside skin. To test possible changes, the skin cross-sections were analysed after the end of the experiment (close to the natural lifetime limit of these mice). There were no observed in the treated skin. Fig. 4 shows an optical (brightfield) view of cross-sections of the skin of mice # 2 and 4 (which had the largest and average response to the treatment as can be seen from Fig. 2) collected from samples treated with cytochalasin B and placebo cream. One can see no abnormal formation inside the skin. It should be noted that the mice used in this study had a semi-compromised immune system. The Jackson laboratories website states that the NU/J strain mice lack T cells (athymic), and therefore, lack cell mediated immunity, but can develop extrathymic T cell function with age. They have normal B cells, and have normal numbers and functions of macrophages, NK cells, and APCs. Therefore, it is unlikely that the inflammation response is affected. One of the main concerns was development of tumours because it is a typical cause of death of these NU/J strain mice. No mouse in the treatment group developed skin rash, inflammation, or cancer during the course of the experiment. At the same time, one mouse in the control group developed cancer near its cheek close the end of the experiment duration. Although it is certainly not enough statistics to make a definite statement, the lack of undesirable skin changes observed in the mice of this study is rather promising.

### In vitro human model

**Fig 5:**
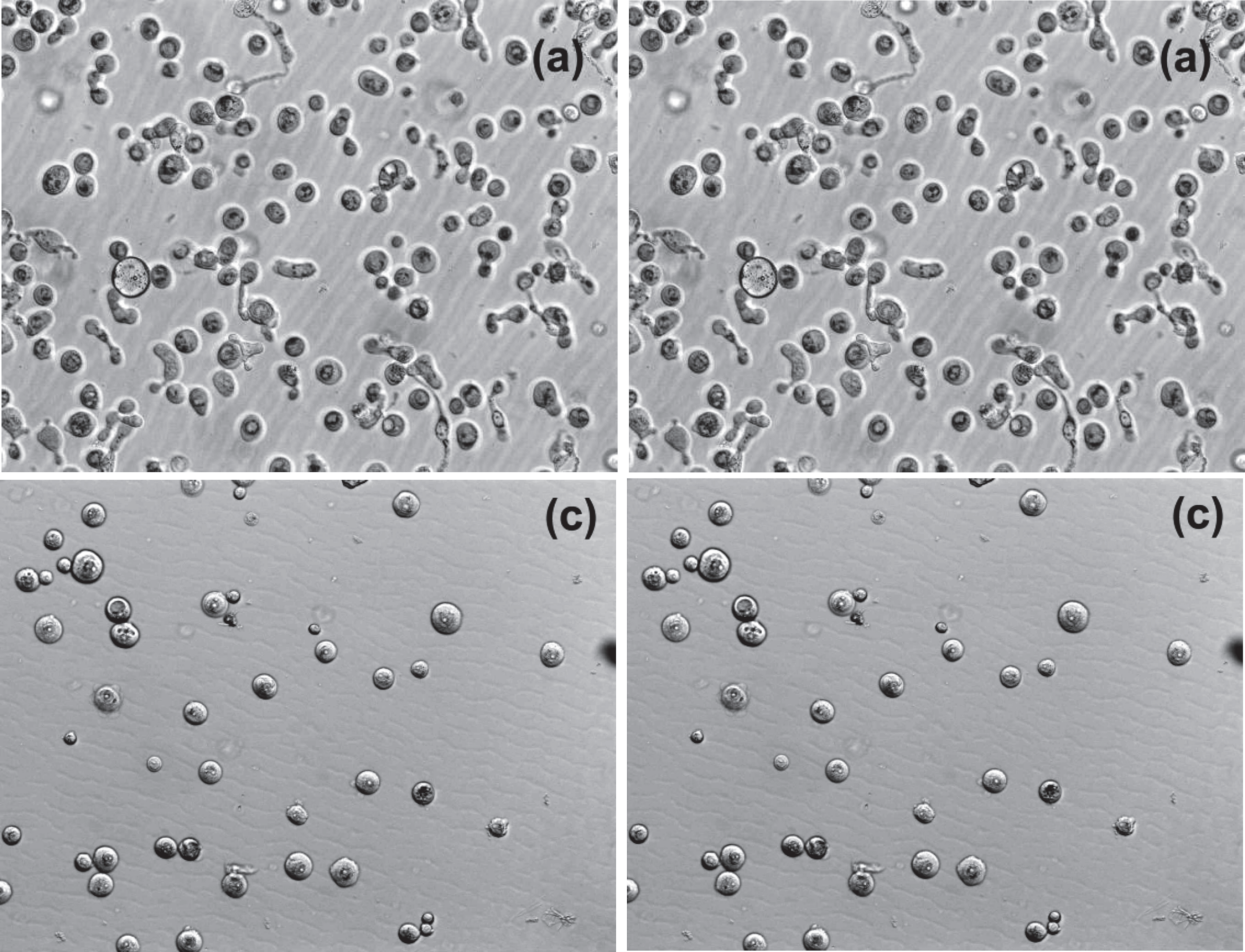
Representative images of cells collected in the Hoffman contrast mode. Images before (left) and after (right) cytochalasin B treatment for passages 2 (a,b) and 6 (c,d).

Fig. 5 shows representative examples of Hoffman optical images of cells. Using this mode, one can obtain unambiguously defined areas of cells (it can depend on the focus when using regular brightfield or phase imaging). Cells at passage number 2 (no change on the cell size) and 6 (the largest cell size change) are shown.

**Fig 6:**
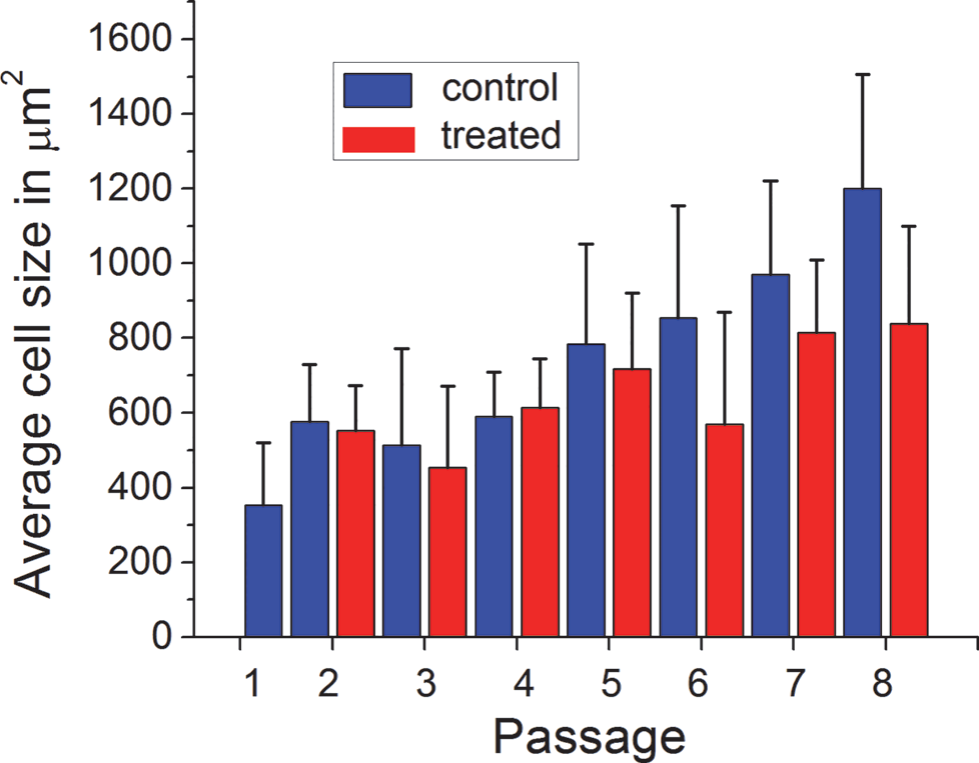
The average size of cells as a function of *in vitro* aging with continued passage. Results for cells without (control) and with the treatment with cytochalasin B are shown. Each “variability” bar corresponds to one standard deviation.

Processing such images to calculate the area of the cells, one can obtain the distribution of cell sizes. Fig. 6 shows the average size of cells as a function of *in vitro* aging, passage 1–8. Passage 8 is close to the end of the cell life. Many cells become visually senescent. Results for untreated cells (control) and treatment with cytochalasin B are shown. The “variability” bar corresponds to one standard deviation. Passage 1 may still carry the stress of cell extraction from tissue, and therefore, was not treated (it is presented for reference only).

One can see from Fig. 6 that the cell size increases with passage. However, the increase is not monotonic, and becomes pronounceable starting from passage 5. If this is associated with the appearance of visual signs of aging, it should correspond to about 50 years of aging *in vivo*. Interestingly, one can see that the cytochalasin treatment starts “working” (become statistically significant at p<0.05) from passage 5. Specifically, the cell size decreases after the cytochalasin treatment.

**Fig 7:**
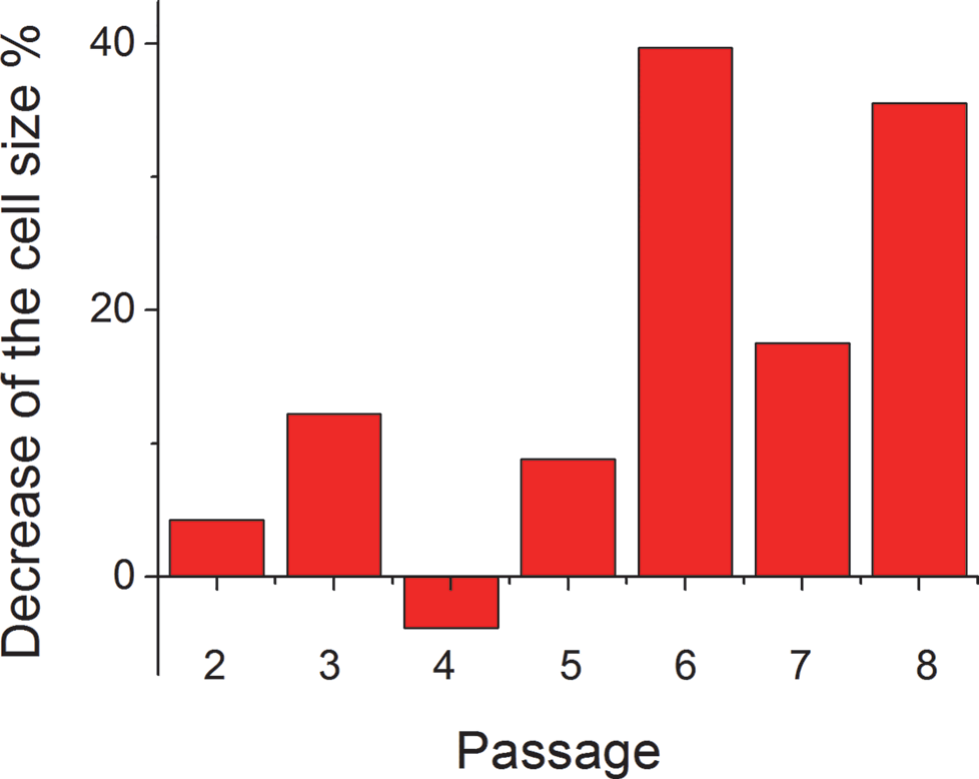
Change in the cell size. The size change is shown after treatment with cytochalasin B relative to the control (Fig. 6).

The difference in the average cell size due to the treatment is shown in Fig. 7 for passages 1–8. Although the variation is quite large, the trend is obvious. Compared to the change observed for mouse cells (56%, Fig. 2), the change is a bit smaller (20–40%) for human cells. Strictly speaking, the quantitative comparison is not entirely correct because of different model (*in vivo* vs *in vitro*), substantially different durations of the treatment (140 days vs 12 hours), and different cell source (mice vs human). Nevertheless, finding a similar behavior of cell response to the cytochalasin treatment is an interesting result. Although it might be plausible to expect the observed decrease by reasoning that the cell is preserving its mechanical integrity by shrinking, the nature of this phenomenon is not still clear. In particular, it is puzzling that younger cells do not change their size after the cytochalasin treatment (as we observed at least *in vitro*).

The nature of the cell response to the treatment reported here is not clear. One problem is the virtual absence of biophysical models to describe aging cells. We could only speculate about the nature of such a cell response. The cell size is presumably defined by a balance between its cytoskeleton, pressure of internal organelles, and the osmotic pressure. As shown previously by using atomic force microscopy [17], the structure of the cortical layer of cellular cytoskeleton changes substantially with the cell age [18]. Specifically, its surface fiber density (cross-linking) substantially increases with age (volume of the cytoskeleton increases as well but not that much). If we assume that the cell size increases in the response to the increase of cytoskeleton microfilaments, the results could be explained as follows. The increase of microfilament densities cytoskeleton presumably starts changing around passage 5. The cell treatment decreases the amount of F-actin (microfilaments), and thus, cells decrease its size. Because the decrease of the amount of F-actin happens for all cells independently of their passage, the dependence of the cell size of the amount of microfilaments should then be non-monotonic. It should not be microfilament-dependent for “thin” cytoskeleton of young cells. Being plausible, the above explanation is still a speculation. A suitable model of cellular aging is yet to be developed. This will be the scope of future works.

## Conclusion

Here we showed that treatment of aging skin epithelial cells with a fungal toxin, cytochalasin B resulted in a substantial decrease in cell size. This was demonstrated using a mouse model *(in vivo*) and on human skin cells *in vitro*. The cell response to cytochalasin B reported here seems to be universal for both mouse and human cells, and occurs both *in vitro* and *in vivo*. The lack of noticeable size change after the treatment of younger cells (observed on human cells *in vitro* passages less than 5) was particularly unexpected. The nature of this behavior is presumably related to breaking the balance between the internal cell pressure and the cytoskeleton. These results may be important for better understanding the aging processes in cells.

The study of histological sections of mouse skin after the end of the experiment (close to the natural lifetime limit of these mice) showed that no abnormalities developed. Thus, from a practical point of view, the observed phenomenon could be used in cosmetics to decrease visual signs of aging skin. From a fundamental point of view, aging of skin may be a model for other organs. Therefore, the reported results may have a broader general interest in the area of biological aging.
